# Supplementary material for: Synthesis, Molecular Docking Study, and Cytotoxic Activity against MCF Cells of New Thiazole–Thiophene Scaffolds
Source: Molecules. 2022 Jul 20;27(14):4639. doi: 10.3390/molecules27144639 (PMC9320749; doi:10.3390/molecules27144639)
Supplement: Supplementary file 1 [file molecules-27-04639-s001.zip › molecules-1770459-supplementary.pdf]

Supporting Informations

Synthesis, Anti-breast Cancer Assessment, and Molecular Docking Study of some New Thiazole Clubbed Thiophene Scaffolds

Sobhi M. Gomha[1]*, Sayed M. Riyadh[2], Bader Huwaimel[3], Mohie E. M. Zayed[4], Magda H. Abdellattif[5]

[1)] Department of Chemistry, Faculty of Science, Islamic University of Madinah, Madinah, 42351, Saudi Arabia. smgomha@iu.edu.sa

[2)] Department of Chemistry, Faculty of Science, Cairo University, Giza, 12613, Egypt, Email: s.m.gomha@cu.edu.eg, riyadh1993@hotmail.com

[3)] Department of Pharmaceutical Chemistry, College of Pharmacy, University of Ha'il, Ha'il 81442, Saudi Arabia, Email: b.huwaimel@uoh.edu.sa

[4)] Department of Chemistry, Faculty of Science, King Abdulaziz University, Jeddah 21589, Saudi Arabia, Email: MZAYED@kau.edu.sa

[5)] Department of Chemistry, College of Science, PO Box 11099, Taif 21944, Saudi Arabia, m.hasan@tu.edu.sa

Correspondence:, smgomha@iu.edu.sa

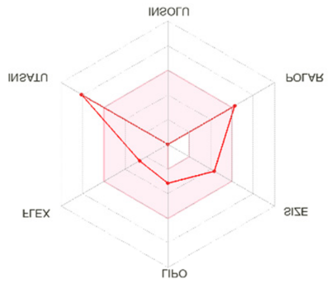

a

4a

Physicochemical Properties	
Formula	C16H16N6S2
Molecular weight	356.47 g/mol
Num. heavy atoms	24
Num. arom. heavy atoms	16
Fraction Csp3	0.12
Num. rotatable bonds	4
Num. H-bond acceptors	4
Num. H-bond donors	1

Molar Refractivity	100.32
TPSA	136.87 Å²
**Lipophilicity**	
Log $P_{o/w}$ (iLOGP)	
Log $P_{o/w}$ (XLOGP3)	
Log $P_{o/w}$ (WLOGP)	4.38
Log $P_{o/w}$ (MLOGP)	
Log $P_{o/w}$ (SILICOS-IT)	
Consensus Log $P_{o/w}$	
**Water Solubility**	
Log S (ESOL)	
Solubility	mg/ml ; mol/l
Class	
Log S (Ali)	
Solubility	mg/ml ; mol/l
Class	
Log S (SILICOS-IT)	
Solubility	mg/ml ; mol/l
Class	
**Pharmacokinetics**	
GI absorption	
BBB permeant	
P-gp substrate	
CYP1A2 inhibitor	
CYP2C19 inhibitor	
CYP2C9 inhibitor	
CYP2D6 inhibitor	
CYP3A4 inhibitor	
Log K_p (skin permeation)	cm/s
**Druglikeness**	
Lipinski	; violation
Ghose	
Veber	
Egan	
Muegge	
Bioavailability Score	

Medicinal Chemistry	
PAINS	0 alert
Brenk	0 alert
Leadlikeness	
Synthetic accessibility	

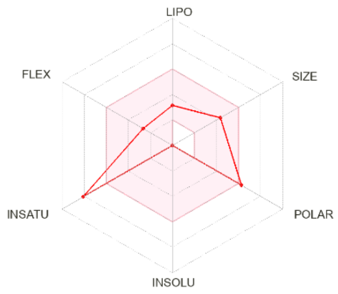

4b

Physicochemical Properties	
Formula	C16H16N6S2
Molecular weight	356.47 g/mol
Num. heavy atoms	24
Num. arom. heavy atoms	16
Fraction Csp3	0.12
Num. rotatable bonds	4
Num. H-bond acceptors	4
Num. H-bond donors	1
Molar Refractivity	100.48
TPSA	136.87 Å²
**Lipophilicity**	
Log $P_{o/w}$ (iLOGP)	
Log $P_{o/w}$ (XLOGP3)	
Log $P_{o/w}$ (WLOGP)	4.30
Log $P_{o/w}$ (MLOGP)	
Log $P_{o/w}$ (SILICOS-IT)	
Consensus Log $P_{o/w}$	
**Water Solubility**	
Log S (ESOL)	
Solubility	mg/ml ; mol/l
Class	
Log S (Ali)	
Solubility	mg/ml ; mol/l

Class	
Log *S* (SILICOS-IT)	
Solubility	mg/ml ; mol/l
Class	
**Pharmacokinetics**	
GI absorption	
BBB permeant	
P-gp substrate	
CYP1A2 inhibitor	
CYP2C19 inhibitor	
CYP2C9 inhibitor	
CYP2D6 inhibitor	
CYP3A4 inhibitor	
Log K_p (skin permeation)	cm/s
**Druglikeness**	
Lipinski	; violation
Ghose	
Veber	
Egan	
Muegge	
Bioavailability Score	
**Medicinal Chemistry**	
PAINS	0 alert
Brenk	0 alert
Leadlikeness	
Synthetic accessibility	

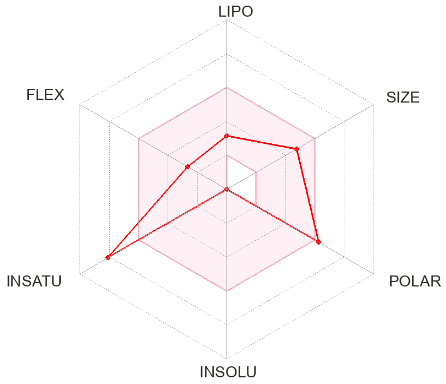

4c

Physicochemical Properties	
Formula	C16H15ClN6S2
Molecular weight	390.91 g/mol
Num. heavy atoms	25
Num. arom. heavy atoms	16
Fraction Csp3	0.12
Num. rotatable bonds	4
Num. H-bond acceptors	4
Num. H-bond donors	1
Molar Refractivity	105.33
TPSA	136.87 Å²
Lipophilicity	
Log $P_{o/w}$ (iLOGP)	
Log $P_{o/w}$ (XLOGP3)	
Log $P_{o/w}$ (WLOGP)	5.04
Log $P_{o/w}$ (MLOGP)	
Log $P_{o/w}$ (SILICOS-IT)	
Consensus Log $P_{o/w}$	
Water Solubility	
Log *S* (ESOL)	
Solubility	mg/ml ; mol/l
Class	
Log *S* (Ali)	
Solubility	mg/ml ; mol/l
Class	
Log *S* (SILICOS-IT)	
Solubility	mg/ml ; mol/l
Class	
Pharmacokinetics	
GI absorption	
BBB permeant	
P-gp substrate	
CYP1A2 inhibitor	
CYP2C19 inhibitor	
CYP2C9 inhibitor	
CYP2D6 inhibitor	
CYP3A4 inhibitor	

Log K_p (skin permeation)	cm/s
Druglikeness	
Lipinski	; violation
Ghose	
Veber	
Egan	
Muegge	
Bioavailability Score	
Medicinal Chemistry	
PAINS	0 alert
Brenk	0 alert
Leadlikeness	
Synthetic accessibility	

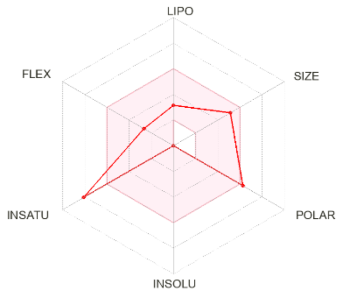

4d

Physicochemical Properties	
Formula	C16H14Cl2N6S2
Molecular weight	425.36 g/mol
Num. heavy atoms	26
Num. arom. heavy atoms	16
Fraction Csp3	0.12
Num. rotatable bonds	4
Num. H-bond acceptors	4
Num. H-bond donors	1
Molar Refractivity	110.34
TPSA	136.87 Å²
Lipophilicity	
Log $P_{o/w}$ (iLOGP)	
Log $P_{o/w}$ (XLOGP3)	
Log $P_{o/w}$ (WLOGP)	5.69
Log $P_{o/w}$ (MLOGP)	
Log $P_{o/w}$ (SILICOS-IT)	

Consensus Log $P_{o/w}$	
**Water Solubility**	
Log *S* (ESOL)	
Solubility	mg/ml ; mol/l
Class	
Log *S* (Ali)	
Solubility	mg/ml ; mol/l
Class	
Log *S* (SILICOS-IT)	
Solubility	mg/ml ; mol/l
Class	
**Pharmacokinetics**	
GI absorption	
BBB permeant	
P-gp substrate	
CYP1A2 inhibitor	
CYP2C19 inhibitor	
CYP2C9 inhibitor	
CYP2D6 inhibitor	
CYP3A4 inhibitor	
Log K_p (skin permeation)	cm/s
**Druglikeness**	
Lipinski	; violation
Ghose	
Veber	
Egan	
Muegge	
Bioavailability Score	
**Medicinal Chemistry**	
PAINS	0 alert
Brenk	0 alert
Leadlikeness	
Synthetic accessibility	

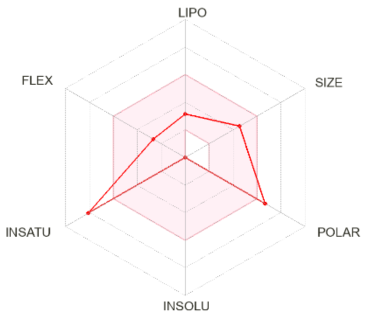

8a

Physicochemical Properties	
Formula	C16H16N6OS2
Molecular weight	372.47 g/mol
Num. heavy atoms	25
Num. arom. heavy atoms	11
Fraction Csp3	0.12
Num. rotatable bonds	4
Num. H-bond acceptors	5
Num. H-bond donors	2
Molar Refractivity	108.56
TPSA	148.98 Å²
**Lipophilicity**	
Log $P_{o/w}$ (iLOGP)	
Log $P_{o/w}$ (XLOGP3)	
Log $P_{o/w}$ (WLOGP)	2.44
Log $P_{o/w}$ (MLOGP)	
Log $P_{o/w}$ (SILICOS-IT)	
Consensus Log $P_{o/w}$	
**Water Solubility**	
Log S (ESOL)	
Solubility	mg/ml ; mol/l
Class	
Log S (Ali)	
Solubility	mg/ml ; mol/l
Class	
Log S (SILICOS-IT)	
Solubility	mg/ml ; mol/l
Class	
**Pharmacokinetics**	

GI absorption	
BBB permeant	
P-gp substrate	
CYP1A2 inhibitor	
CYP2C19 inhibitor	
CYP2C9 inhibitor	
CYP2D6 inhibitor	
CYP3A4 inhibitor	
Log K_p (skin permeation)	cm/s
**Druglikeness**	
Lipinski	; violation
Ghose	
Veber	
Egan	
Muegge	
Bioavailability Score	
**Medicinal Chemistry**	
PAINS	0 alert
Brenk	0 alert
Leadlikeness	
Synthetic accessibility	

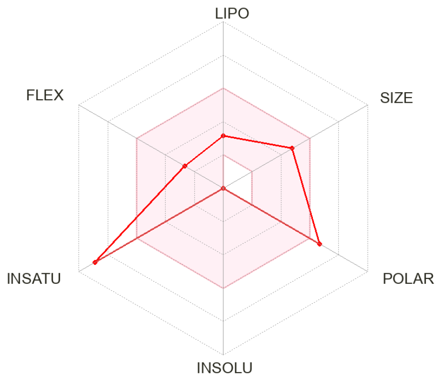

8b

Physicochemical Properties	
Formula	C15H13ClN6OS2
Molecular weight	392.89 g/mol
Num. heavy atoms	25
Num. arom. heavy atoms	11
Fraction Csp3	0.07
Num. rotatable bonds	4

Num. H-bond acceptors	5
Num. H-bond donors	2
Molar Refractivity	108.60
TPSA	148.98 Å²
Lipophilicity	
Log $P_{o/w}$ (iLOGP)	
Log $P_{o/w}$ (XLOGP3)	
Log $P_{o/w}$ (WLOGP)	2.78
Log $P_{o/w}$ (MLOGP)	
Log $P_{o/w}$ (SILICOS-IT)	
Consensus Log $P_{o/w}$	
Water Solubility	
Log *S* (ESOL)	
Solubility	mg/ml ; mol/l
Class	
Log *S* (Ali)	
Solubility	mg/ml ; mol/l
Class	
Log *S* (SILICOS-IT)	
Solubility	mg/ml ; mol/l
Class	
Pharmacokinetics	
GI absorption	
BBB permeant	
P-gp substrate	
CYP1A2 inhibitor	
CYP2C19 inhibitor	
CYP2C9 inhibitor	
CYP2D6 inhibitor	
CYP3A4 inhibitor	
Log K_p (skin permeation)	cm/s
Druglikeness	
Lipinski	; violation
Ghose	
Veber	
Egan	
Muegge	

Bioavailability Score	
**Medicinal Chemistry**	
PAINS	0 alert
Brenk	0 alert
Leadlikeness	
Synthetic accessibility	

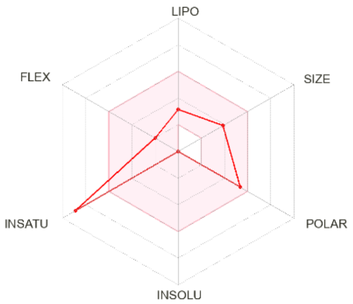

11a

Physicochemical Properties	
Formula	C15H14N4S2
Molecular weight	314.43 g/mol
Num. heavy atoms	21
Num. arom. heavy atoms	16
Fraction Csp3	0.07
Num. rotatable bonds	3
Num. H-bond acceptors	2
Num. H-bond donors	1
Molar Refractivity	90.17
TPSA	112.15 Å²
**Lipophilicity**	
Log $P_{o/w}$ (iLOGP)	
Log $P_{o/w}$ (XLOGP3)	
Log $P_{o/w}$ (WLOGP)	3.32
Log $P_{o/w}$ (MLOGP)	
Log $P_{o/w}$ (SILICOS-IT)	
Consensus Log $P_{o/w}$	
**Water Solubility**	
Log S (ESOL)	
Solubility	mg/ml ; mol/l
Class	
Log S (Ali)	

Solubility	mg/ml ; mol/l
Class	
Log *S* (SILICOS-IT)	
Solubility	mg/ml ; mol/l
Class	
**Pharmacokinetics**	
GI absorption	
BBB permeant	
P-gp substrate	
CYP1A2 inhibitor	
CYP2C19 inhibitor	
CYP2C9 inhibitor	
CYP2D6 inhibitor	
CYP3A4 inhibitor	
Log K_p (skin permeation)	cm/s
**Druglikeness**	
Lipinski	; violation
Ghose	
Veber	
Egan	
Muegge	
Bioavailability Score	
**Medicinal Chemistry**	
PAINS	0 alert
Brenk	0 alert
Leadlikeness	
Synthetic accessibility	

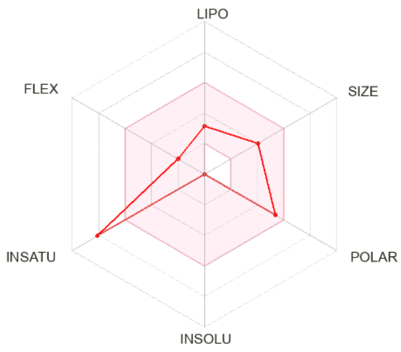

11b

Physicochemical Properties

Formula	C16H16N4S2
Molecular weight	328.46 g/mol
Num. heavy atoms	22
Num. arom. heavy atoms	16
Fraction Csp3	0.12
Num. rotatable bonds	3
Num. H-bond acceptors	2
Num. H-bond donors	1
Molar Refractivity	95.14
TPSA	112.15 Å²
**Lipophilicity**	
Log $P_{o/w}$ (iLOGP)	
Log $P_{o/w}$ (XLOGP3)	
Log $P_{o/w}$ (WLOGP)	3.63
Log $P_{o/w}$ (MLOGP)	
Log $P_{o/w}$ (SILICOS-IT)	
Consensus Log $P_{o/w}$	
**Water Solubility**	
Log S (ESOL)	
Solubility	mg/ml ; mol/l
Class	
Log S (Ali)	
Solubility	mg/ml ; mol/l
Class	
Log S (SILICOS-IT)	
Solubility	mg/ml ; mol/l
Class	
**Pharmacokinetics**	
GI absorption	
BBB permeant	
P-gp substrate	
CYP1A2 inhibitor	
CYP2C19 inhibitor	
CYP2C9 inhibitor	
CYP2D6 inhibitor	
CYP3A4 inhibitor	
Log K_p (skin permeation)	cm/s

Druglikeness	
Lipinski	; violation
Ghose	
Veber	
Egan	
Muegge	
Bioavailability Score	
**Medicinal Chemistry**	
PAINS	0 alert
Brenk	0 alert
Leadlikeness	
Synthetic accessibility	

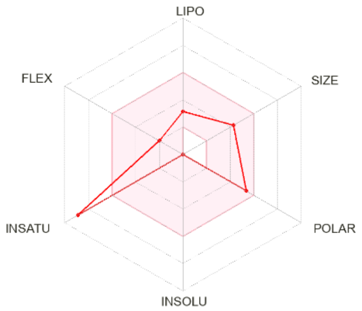

11c

Physicochemical Properties	
Formula	C15H13ClN4S2
Molecular weight	348.87 g/mol
Num. heavy atoms	22
Num. arom. heavy atoms	16
Fraction Csp3	0.07
Num. rotatable bonds	3
Num. H-bond acceptors	2
Num. H-bond donors	1
Molar Refractivity	95.18
TPSA	112.15 Å^2
**Lipophilicity**	
Log $P_{o/w}$ (iLOGP)	
Log $P_{o/w}$ (XLOGP3)	
Log $P_{o/w}$ (WLOGP)	3.98
Log $P_{o/w}$ (MLOGP)	
Log $P_{o/w}$ (SILICOS-IT)	
Consensus Log $P_{o/w}$	

**Water Solubility**	
Log *S* (ESOL)	
Solubility	mg/ml ; mol/l
Class	
Log *S* (Ali)	
Solubility	mg/ml ; mol/l
Class	
Log *S* (SILICOS-IT)	
Solubility	mg/ml ; mol/l
Class	
**Pharmacokinetics**	
GI absorption	
BBB permeant	
P-gp substrate	
CYP1A2 inhibitor	
CYP2C19 inhibitor	
CYP2C9 inhibitor	
CYP2D6 inhibitor	
CYP3A4 inhibitor	
Log K_p (skin permeation)	cm/s
**Druglikeness**	
Lipinski	; violation
Ghose	
Veber	
Egan	
Muegge	
Bioavailability Score	
**Medicinal Chemistry**	
PAINS	0 alert
Brenk	0 alert
Leadlikeness	
Synthetic accessibility	

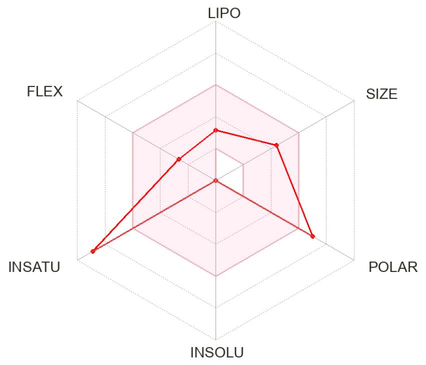

11d

**physicochemical Properties**	
Formula	C15H13N5O2S2
Molecular weight	359.43 g/mol
Num. heavy atoms	24
Num. arom. heavy atoms	16
Fraction Csp3	0.07
Num. rotatable bonds	4
Num. H-bond acceptors	4
Num. H-bond donors	1
Molar Refractivity	98.99
TPSA	157.97 Å^2
**Lipophilicity**	
Log $P_{o/w}$ (iLOGP)	
Log $P_{o/w}$ (XLOGP3)	
Log $P_{o/w}$ (WLOGP)	3.23
Log $P_{o/w}$ (MLOGP)	
Log $P_{o/w}$ (SILICOS-IT)	
Consensus Log $P_{o/w}$	
**Water Solubility**	
Log S (ESOL)	
Solubility	mg/ml ; mol/l
Class	
Log S (Ali)	
Solubility	mg/ml ; mol/l
Class	
Log S (SILICOS-IT)	
Solubility	mg/ml ; mol/l
Class	
**Pharmacokinetics**	

GI absorption	
BBB permeant	
P-gp substrate	
CYP1A2 inhibitor	
CYP2C19 inhibitor	
CYP2C9 inhibitor	
CYP2D6 inhibitor	
CYP3A4 inhibitor	
Log K_p (skin permeation)	cm/s
**Druglikeness**	
Lipinski	; violation
Ghose	
Veber	
Egan	
Muegge	
Bioavailability Score	
**Medicinal Chemistry**	
PAINS	0 alert
Brenk	0 alert
Leadlikeness	
Synthetic accessibility	

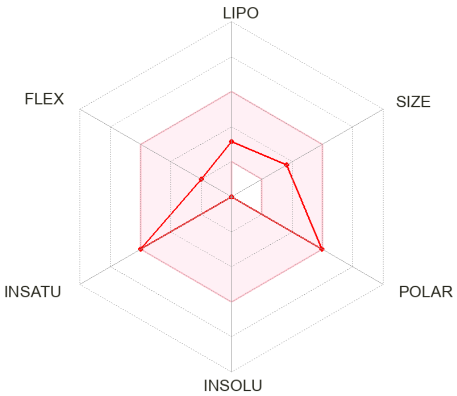

14a

**Physicochemical Properties**	
Formula	C12H14N4OS2
Molecular weight	294.40 g/mol
Num. heavy atoms	19
Num. arom. heavy atoms	10
Fraction Csp3	0.25
Num. rotatable bonds	3

Num. H-bond acceptors	3
Num. H-bond donors	1
Molar Refractivity	79.89
TPSA	129.22 Å²
Lipophilicity	
Log $P_{o/w}$ (iLOGP)	
Log $P_{o/w}$ (XLOGP3)	
Log $P_{o/w}$ (WLOGP)	2.17
Log $P_{o/w}$ (MLOGP)	
Log $P_{o/w}$ (SILICOS-IT)	
Consensus Log $P_{o/w}$	
Water Solubility	
Log S (ESOL)	
Solubility	mg/ml ; mol/l
Class	
Log S (Ali)	
Solubility	mg/ml ; mol/l
Class	
Log S (SILICOS-IT)	
Solubility	mg/ml ; mol/l
Class	
Pharmacokinetics	
GI absorption	
BBB permeant	
P-gp substrate	
CYP1A2 inhibitor	
CYP2C19 inhibitor	
CYP2C9 inhibitor	
CYP2D6 inhibitor	
CYP3A4 inhibitor	
Log K_p (skin permeation)	cm/s
Druglikeness	
Lipinski	; violation
Ghose	
Veber	
Egan	
Muegge	

Bioavailability Score	
Medicinal Chemistry	
PAINS	0 alert
Brenk	0 alert
Leadlikeness	
Synthetic accessibility	

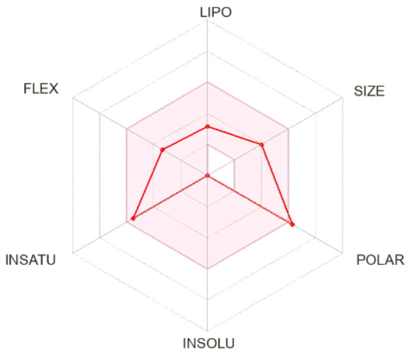

14b

Physicochemical Properties	
Formula	C13H16N4O2S2
Molecular weight	324.42 g/mol
Num. heavy atoms	21
Num. arom. heavy atoms	10
Fraction Csp3	0.31
Num. rotatable bonds	5
Num. H-bond acceptors	4
Num. H-bond donors	1
Molar Refractivity	85.79
TPSA	138.45 Å²
Lipophilicity	
Log $P_{o/w}$ (iLOGP)	
Log $P_{o/w}$ (XLOGP3)	
Log $P_{o/w}$ (WLOGP)	2.14
Log $P_{o/w}$ (MLOGP)	
Log $P_{o/w}$ (SILICOS-IT)	
Consensus Log $P_{o/w}$	
Water Solubility	
Log S (ESOL)	
Solubility	mg/ml ; mol/l
Class	

Log *S* (Ali)	
Solubility	mg/ml ; mol/l
Class	
Log *S* (SILICOS-IT)	
Solubility	mg/ml ; mol/l
Class	
**Pharmacokinetics**	
GI absorption	
BBB permeant	
P-gp substrate	
CYP1A2 inhibitor	
CYP2C19 inhibitor	
CYP2C9 inhibitor	
CYP2D6 inhibitor	
CYP3A4 inhibitor	
Log K_p (skin permeation)	cm/s
**Druglikeness**	
Lipinski	; violation
Ghose	
Veber	
Egan	
Muegge	
Bioavailability Score	
**Medicinal Chemistry**	
PAINS	0 alert
Brenk	0 alert
Leadlikeness	
Synthetic accessibility	

Figure S1: Extra ADME results
